# A global cloud free pixel- based image composite from Sentinel-2 data

**DOI:** 10.1016/j.dib.2020.105737

**Published:** 2020-05-21

**Authors:** C. Corbane, P. Politis, P. Kempeneers, D. Simonetti, P. Soille, A. Burger, M. Pesaresi, F. Sabo, V. Syrris, T. Kemper

**Affiliations:** a European Commission, Joint Research Centre; b Arhs Developments S.A., 4370, Belvaux, Luxembourg

**Keywords:** Pixel based composite, Sentinel-2 satellite, land cover classification, large area mapping, remote sensing

## Abstract

Large-scale land cover classification from satellite imagery is still a challenge due to the big volume of data to be processed, to persistent cloud-cover in cloud-prone areas as well as seasonal artefacts that affect spatial homogeneity. Sentinel-2 times series from Copernicus Earth Observation program offer a great potential for fine scale land cover mapping thanks to high spatial and temporal resolutions, with a decametric resolution and five-day repeat time. However, the selection of best available scenes, their download together with the requirements in terms of storage and computing resources pose restrictions for large-scale land cover mapping. The dataset presented in this paper corresponds to global cloud-free pixel based composite created from the Sentinel-2 data archive (Level L1C) available in Google Earth Engine for the period January 2017- December 2018. The methodology used for generating the image composite is described and the metadata associated with the 10 m resolution dataset is presented. The data with a total volume of 15 TB is stored on the Big Data platform of the Joint Research Centre. It can be downloaded per UTM grid zone, loaded into GIS clients and displayed easily thanks to pre-computed overviews.

Specifications table

**Table:** 

Subject	Earth and Planetary Sciences (General)
Specific subject area	Remote sensing in general and land cover classification in particular
Type of data	Satellite image, tables, figures
How data were acquired	Computed in and exported from Google Earth Engine
Data format	Raw Analyzed Filtered
Parameters for data collection	Cloud coverage limited to 2% (30% in cloud-prone areas such as tropical areas). Observation period from 01/01/2017 until 12/31/2018
Description of data collection	Data queried, analysed and processed in Google earth Engine from L1C Sentinel 2A and 2B
Data source location	Global data covering all landmass Spatial extent (in Pseudo-Mercator, WGS 84, EPSG: 4326): Upper Left 175d15′ 4.36"W, 84d 3′54.78"N Lower Right: 175d17′ 5.59"E, 56d17′52.49"S Projection: Local UTM projection
Data accessibility	Repository name: Joint Research Centre Data Catalogue Data identification number: doi:10.2905/0BD1DFAB-E311-4046-8911-C54A8750DF79 PID: http://data.europa.eu/89h/0bd1dfab-e311-4046-8911-c54a8750df79 https://data.jrc.ec.europa.eu/dataset/0bd1dfab-e311-4046-8911-c54a8750df79

## Value of the data

• The global 10 meters resolution composite from Sentinel-2 images facilitates the automation of large-scale image classification tasks. It has been used for instance as the core input data for the update of the Global Human Settlement Layer data by mapping built-up areas at a spatial resolution of 10 meters.
• Any remote sensing scientists or image analyst can benefit from this generic data that supports large-scale land cover classification tasks including also photo-interpretation and validation tasks.
• Although only 4 out of the 13 Sentinel-2 bands were used to generate the composite at 10 meter resolution (Red, Green, Blue and Near Infra-red) the composite can be used for deriving indices such as the Normalized Difference Vegetation Index and other spatial features typically used as inputs to automatic land cover classification.
• The dataset solves the problem of data selection, filtering, download and storage of Sentinel-2 imagery. The composite allows circumventing these issues by condensing the information in one layer that is cloud-free, radiometrically consistent and spatially contiguous. The composite rule applied ensures a true representation of the data acquired, without spatial or spectral smoothing.

## 1 Data Description

**Figure 1:**
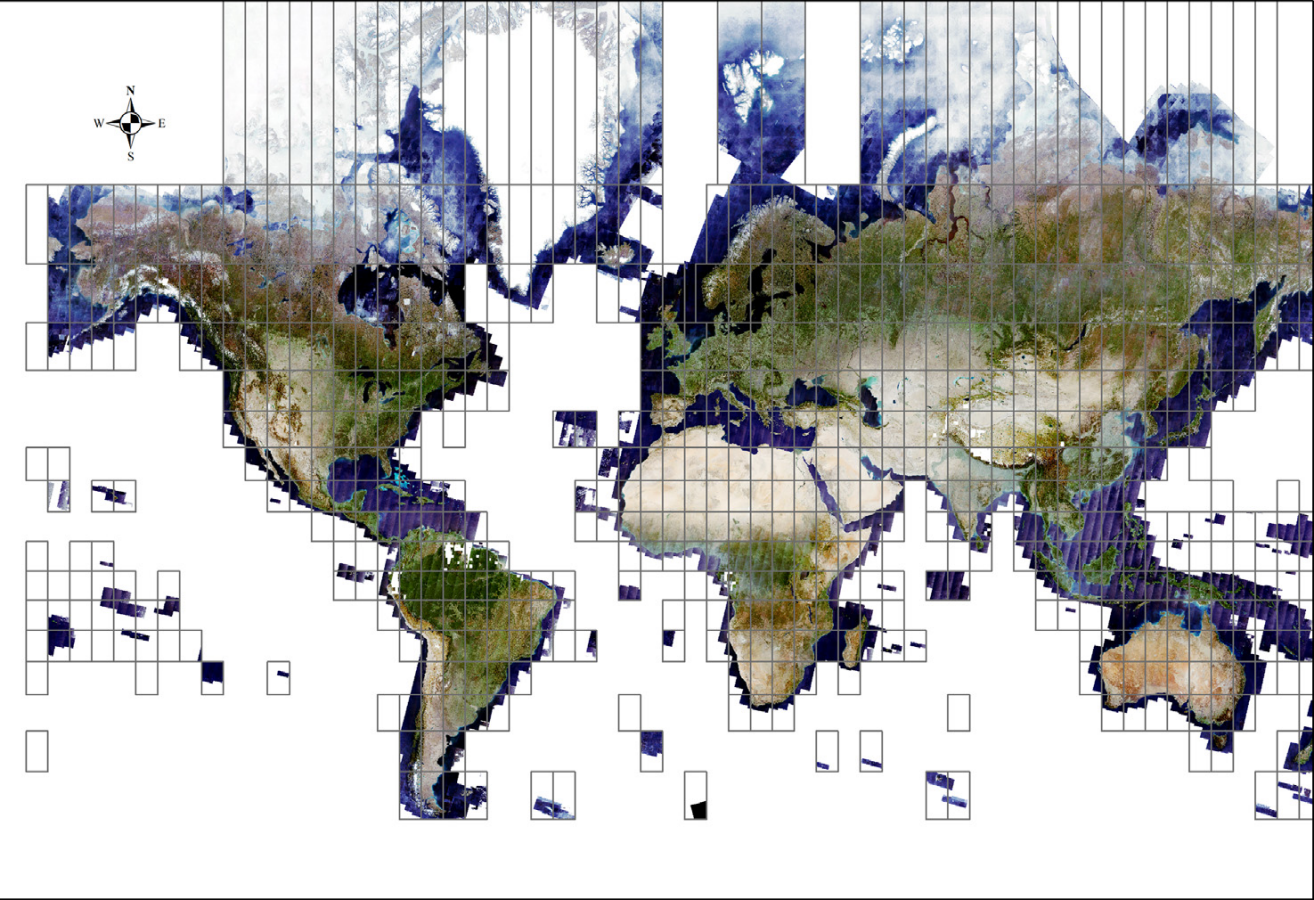
Overview of the cloud free Sentinel-2 image composite organized by UTM grid zone.

The pixel based image composite consists of a global scale raster grid of four spectral bands (B2: Blue, B3: Green, B4: Red and B8: Near Infrared) with a spatial resolution of 10 meters. It was produced and tiled following the Universal Transverse Mercator (UTM) system with each tile having the projection of the UTM zones (UTM/WGS84 projection) to which it corresponds to. There are in total 615 grid zones with data covering mostly mainland and islands. The user has the possibility to download the data per UTM grid zone by selecting the code of the UTM grid zone (e.g. 10U) (Fig. 1). The full dataset has a total volume of 15 TB and is hosted on the Joint Research Centre Big Data platform (JEODPP) [1]. The raster data is stored in 16-bit geotiff format. It can be freely accessed and downloaded from the Open Data Catalogue of the Joint Research Centre of the European Commission [2].

**Figure 2:**
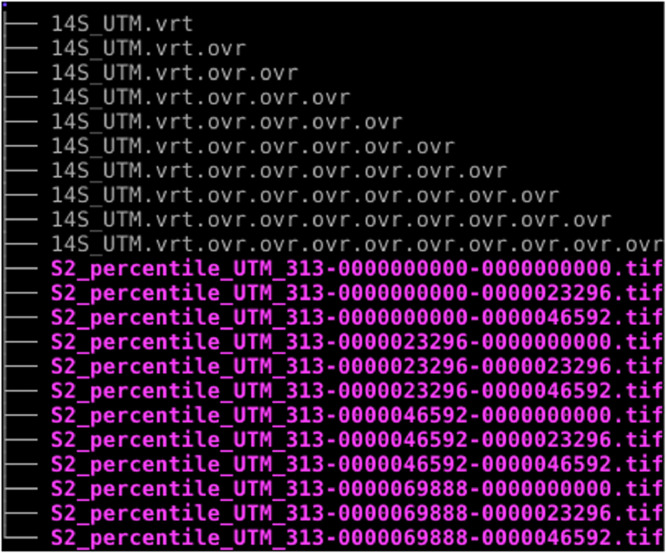
Tree structure of the content of a downloadable folder for a selected UTM grid zone (i.e. 14S).

**Figure 3:**
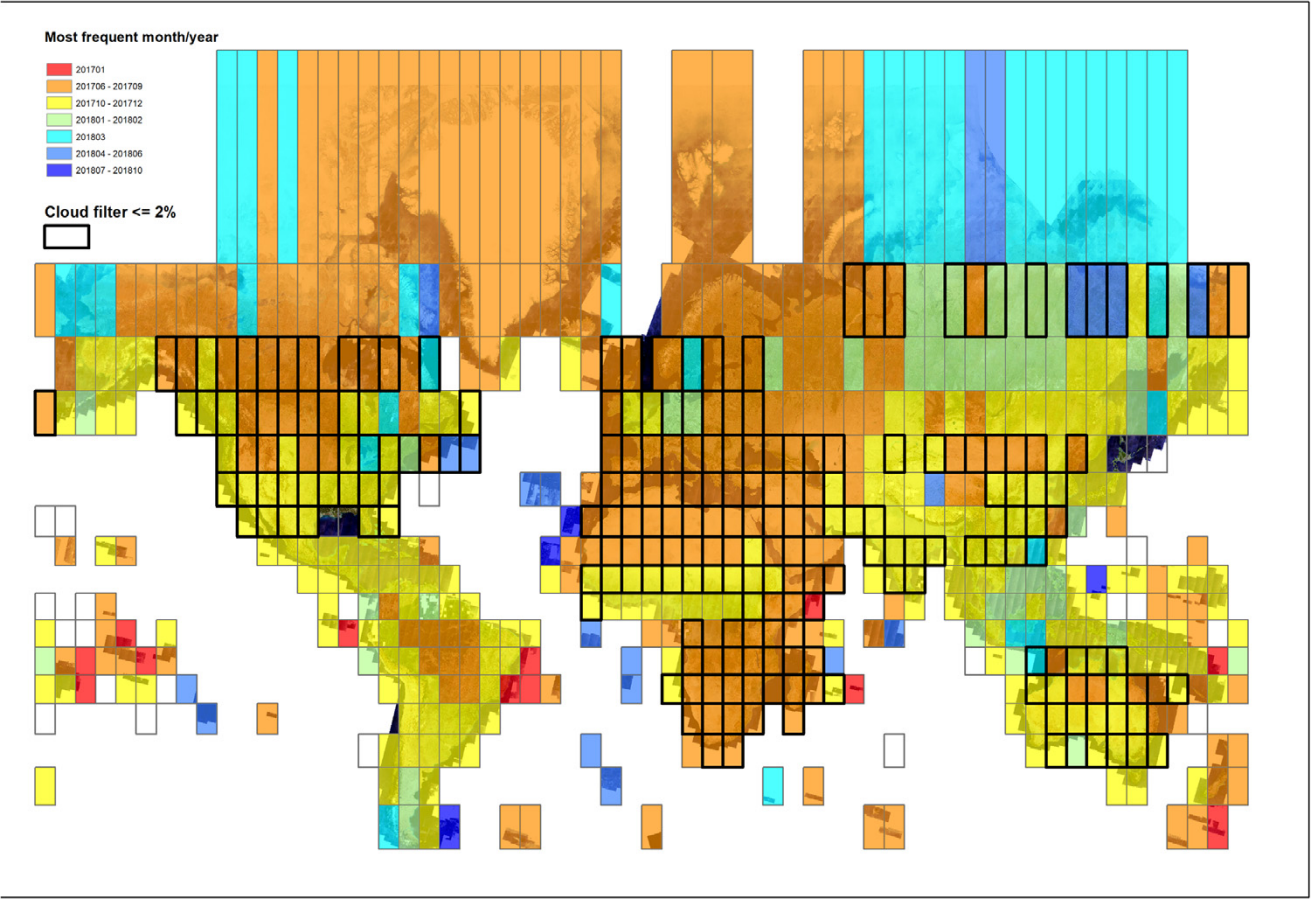
Map showing the most frequent month and year (aggregated per UTM grid zone) of the selected Sentinel-2 images contributing to the generation of the composite.

To facilitate the export and further processing of the composite from GEE, the raster grid per UTM grid zones was sub-tiled into small GeoTIFF files of an average size of 2 GB. Each sub-tile consists of a four band image (Band 1: Red, Band 2: Green, Band 3: Blue and Band 4: Near Infra-red) encoded in 16 bit unsigned integer (uint16). Virtual rasters (.vrt) are created to manage the multiple GeoTIFF files within each UTM grid zone. Virtual rasters are xml files that point to the actual GeoTIFF files. They are useful because they allow handling of large datasets as if they were a single file. Pyramid files (.ovr) at 9 zoom levels are generated for each UTM grid zone to speed up the display of the raster data. When downloading the composite for a selected UTM grid zone, the user is expected to get a set of files as in this example (Fig. 2):

In addition to the raster data, metadata on the most frequent month of the year of the Sentinel-2 images that were selected for generating the composite, aggregated per UTM grid zone, are also provided in the form of a shapefile (.shp) (Fig. 3).

## 2 Experimental Design, Materials, and Methods

In the field of remote sensing, pixel-based image compositing is an approach that allows to overcome the limitations related to data availability, cloud-coverage, discontinuity in image archives, atmospheric interference and radiometric inconsistencies due to seasonal differences or changes in sun angles [3,4]. Free, full and open access to Sentinel-2 data enables the generation of pixel-based composites that can be used for a broad range of applications with a large spatial coverage such as land cover mapping with supervised or unsupervised classifications methods [5]. The Sentinel-2 mission of the European Copernicus Earth observation program has become operational in October 2017 providing time series of images with a free, full and open access policy with the following characteristics: 13 spectral bands from 0.44–2.2 μm, high spatial resolution images (between 10 m and 60 m depending on the spectral bands), steady and frequent observations. The constellation of Sentinel-2A and -2B satellites observe Earth's land surfaces with a 5-day repeat cycle. [6]. They daily generate about 1.6 TB of compressed raw image data (Drusch et al., 2012). With such characteristics, Sentinel-2 has performed acquisitions above each land pixel at least every five days since it became operational in 2017. Nevertheless, the management of massive amounts of satellite data comes at a cost, both technically and financially. The need for storage and processing infrastructures combined with the time required for exploiting the results may hinder the usage of this data. To circumvent such technical bottlenecks and other restrictions related to data availability, pixel-based image compositing, a well-known technique [7], is proposed as a solution.

Currently, the new Sentinel-2 Global Mosaic service (S2GM) of the Copernicus Global Land Service is providing on-demand composites from time series of Sentinel-2 surface reflectance (level 2A) observations from different compositing periods ranging from one day to one year on a global scale [8]. However, the algorithms used for the image compositing rely on the scene classification of Sentinel-2 L2A data, which is prone to errors (e.g. confusion between clouds and high reflectance built-up areas) which eventually result in undesired artefacts in the S2GM products.

Kempeneers et al. [9] proposed a novel method for image compositing and produced a global composite from Sentinel-2 data on the basis of an optimization scheme that selects a subset of the Sentinel-2 archive in order to reduce the amount of processing. Their method is applicable to situations where data download storage and processing constraints represent a limiting factor. However, the selection process that is based on quicklooks and the cloud mask of Level 1C original images, combined with the setting of the maximum number of overlapping image tiles to 5, resulted in a global composite contaminated with persistent cloud coverage and shadows limiting its usage for large scale classification purposes.

Simonetti et al., [10] proposed a Sentinel-2 Level 1C pan-tropical composite for 2017 and 2018 by extracting per-band annual median values after cloud and shadow masking based on spectral conditions specifically developed for tropical regions. The approach is promising for generating annual cloud-free composites and is currently being adapted for a deployment at global scale.

Recently, the availability of cloud-computing infrastructures hosting entire archives of remote sensing data such as Landsat, Sentinel-1 and Sentinel-2 and offering processing capabilities (e.g. DIAS, AWS, Google Earth Engine), allows overcoming the limitations related to the selection, download and storage of raw data for further processing. The Google Earth Engine (GEE), in particular, provides free access to Sentinel-2 archives and large-scale analysis capabilities for scientific applications.

The dataset presented in this paper exploits these benefits offered by the GEE platform that enabled the development of a cloud-free pixel based composite created from the Sentinel-2 data archive (Level L1C) available in this platform for the period January 2017- December 2018.

### 2.1 Selection of Sentinel-2 tiles and image compositing approach

Input to the compositing process are top of atmosphere Sentinel-2 image tiles, from the so-called Level 1C (L1C) product available in the GEE as an image satellite collection from 23/06/2015 until present. Each Sentinel-2 product may contain multiple granules. The Sentinel-2 data contain 13 uint16 spectral bands representing TOA reflectance scaled by 10 000 [11]. In addition, three QA bands are present where one (QA60) is a bitmask band with cloud mask information. To convert to floating point, the values should be divided by 10 000.

The first step in image compositing is to define the desired time frame for which a cloud-free image is to be created. Usually the image composition is performed for a specific season (e.g. 2-4 months) within a predefined year to reduce the effect of phenology (i.e. seasonal variation of the land cover types). However, given the need to generate a global composite, data gaps can be expected in areas with persistent cloud coverage throughout the seasons in heavily clouded regions. One approach to improve the composite is to define extended time frames (e.g. one year) by including multi-temporal images from the same season of the previous (or following) years. For the global Sentinel-2 cloud-free composite, the reference year was set to 2018 with an extended period covering the year 2017.

The second step, is to select the least cloudy Sentinel-2 granules by filtering the image collection acquired in the predefined time frame on the basis of the percentage of cloud coverage. The Q60 band with cloud information embedded in the L1C Sentinel-2 images was used to pre-filter the image collection. For almost 35 % of the UTM grid zones, a threshold of 2% for the cloud coverage was sufficient to generate cloud-free composite in the UTM grid zone. For the other 65% of UTM zones, the cloud threshold had to be increased to 30% to avoid no data gaps due to clouds (see Fig. 3, showing in thick outline the UTM grid zones where the 2% cloud threshold rule has been applied).

Once the images have been selected, the last step is to composite all observations in time per UTM grid zones. The composite algorithm selects the best pixel observations in time, depending on statistical characteristics of the reflectance values. Several methods have been tested (e.g. the scoring of each pixel on the basis of cloud probability and different indices like brightness, mean value of bands, NDVI and NDSI [12]) but none produced a globally consistent cloud and shadow free composite. The approach based on computing the distribution of all selected observations in a UTM grid zone and by assigning, to the composite image, the pixel value which is the 25^th^ percentile of least cloudy pixels, produced the best quality cloud–free composite on the basis of visual inspection by trained image analysts. The choice of the 25^th^ percentile is robust to outliers and extreme values introduced by cloudy pixels or cloud shadows.

**Figure 4:**
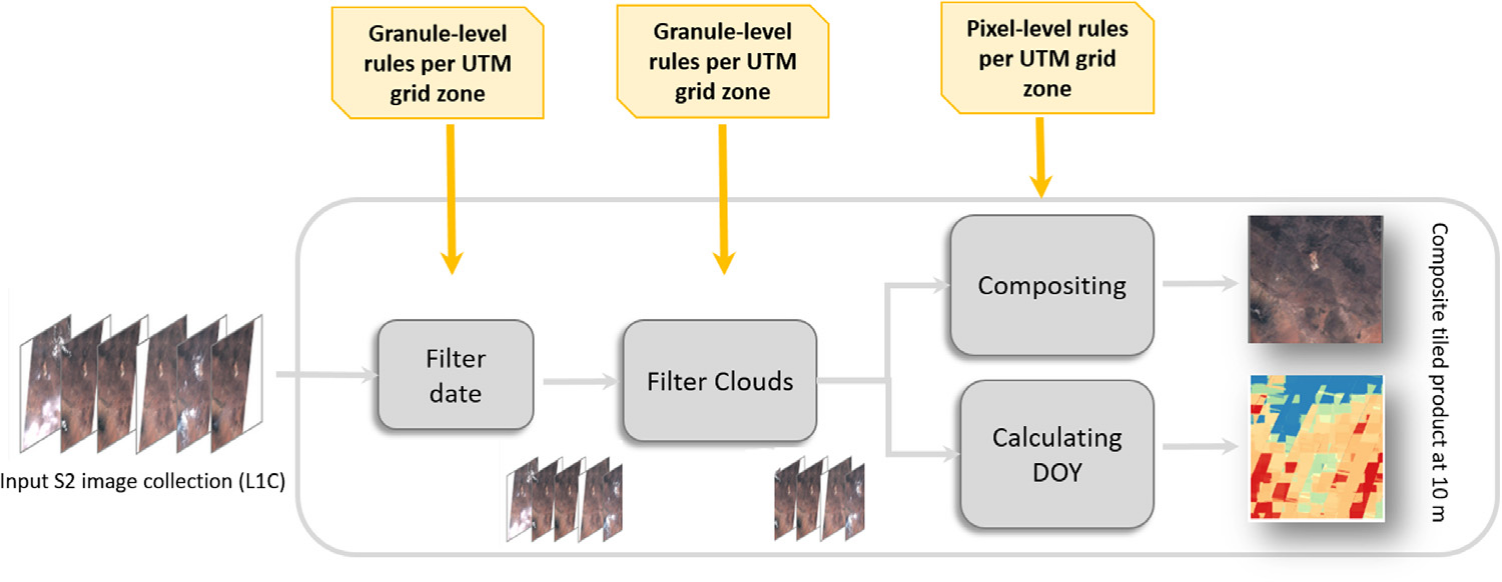
Overview of the image selection and compositing workflow.

The compositing algorithm is applied only to the four 10 meter bands of the Sentinel-2 as a compromise between the need to compute and export the composite at global scale without exceeding the GEE user quotas and the requirements for land cover classification at high spatial resolution. A simplified representation of the compositing workflow is given in Fig. 4. All processing steps including the date and cloud filtering and the compositing, are performed per UTM grid zone. This approach, in combination with the extended time frame covering 2017 and 2018 for the selection of the images, increased the likelihood for the pixels to come from the same season, hence reducing phenology changes within the same UTM grid zone, while avoiding data gaps and reducing the probability of cloud coverage.

The quality of the global composite is strongly related to the quality of the official Sentinel-2 L1C images used as input and provided by the Copernicus service. Issues related to temporal registration or cross-sensor registration for Sentinel-2A and -2B as well as incorrect cloud coverage metadata may affect locally the quality of the composite.

### 2.2 Extraction of metadata on date of each image contributing to the composite

**Figure 5:**
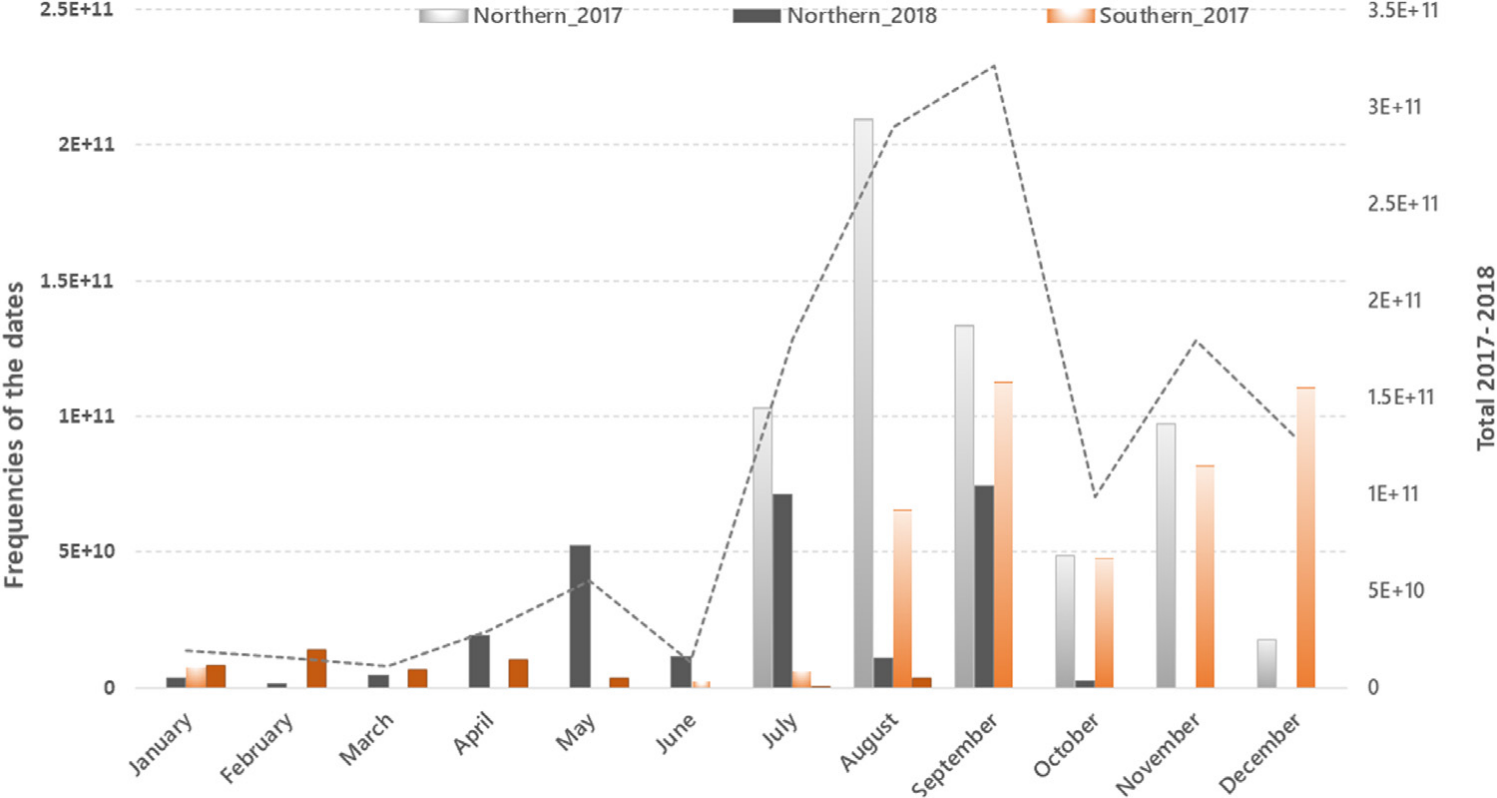
Frequency of the days of the year (calculated at pixel level), aggregated to the month-year, of the images selected for generating the global 10 m resolution composite.

In addition to the cloud-free pixel based composite, a raster grid at 10 meter resolution was also computed representing the Day of the Year (DOY) of the images contributing the composite (Fig. 3). To compute such a raster grid, a function that adds the DOY as band was applied to each image of the filtered Sentinel-2 collection, prior to the application of compositing algorithm. As a result, it was possible to obtain the DOY per pixel of the composite at 10 m resolution. This metadata layer is used here for computing the number of unique dates and the most frequent month-year of the least cloudy granules contributing to the composite. Fig. 5 shows the frequency of the dates of the images selected for generating the composite, split per year (2017 and 2018) and per hemisphere (Northern and Southern) and simplified to the month-year.

The chart shows that most of the images contributing to the final composite were acquired in the summer followed by the fall seasons in the Northern hemisphere whereas in the Southern hemisphere the majority of the images were acquired in spring followed by summer. The images from 2017 were dominant between July and December while those acquired in 2018 were dominant between April and September.

To facilitate the understanding and the handling of the metadata layer, the statistics of the most frequent month-year were aggregated per UTM grid zones and provided together with the global composite in the form of a shapefile (.shp). The attribute table includes for each UTM grid zone, the unique dates expressed as year-month (e.g. 201706) and their corresponding total number of pixels.

## Declaration of Competing Interest

The authors declare that they have no known competing financial interests or personal relationships that could have appeared to influence the work reported in this paper.

## Appendix Supplementary materials

Image, application 1
